# Characteristics of Neonates with Sepsis Associated with Antimicrobial Resistance and Mortality in a Tertiary Hospital in Mexico: A Retrospective Observational Study

**DOI:** 10.3390/pathogens14060588

**Published:** 2025-06-14

**Authors:** Uriel A. Angulo-Zamudio, Maria Luisa Velazquez-Meza, Jesus J. Martinez-Garcia, Nidia Leon-Sicairos, Jorge Velazquez-Roman, Hector Flores-Villaseñor, Claudia Leon-Sicairos, Francisco A. Martínez-Villa, Adrian Canizalez-Roman

**Affiliations:** 1 School of Medicine, Autonomous University of Sinaloa, Culiacan 80019, Sinaloa, Mexico; uriel.zamudio@uas.edu.mx (U.A.A.-Z.); jjmtz64@hotmail.com (J.J.M.-G.); nidialeon@uas.edu.mx (N.L.-S.); jorgevelazquezroman@uas.edu.mx (J.V.-R.); floreshector@uas.edu.mx (H.F.-V.); 2 The Women’s Hospital, Secretariat of Health, Culiacan 80020, Sinaloa, Mexico; marialuisa.velazquez25@gmail.com; 3 Pediatric Hospital of Sinaloa, Culiacan 80200, Sinaloa, Mexico; 4 The Sinaloa State Public Health Laboratory, Secretariat of Health, Culiacan 80020, Sinaloa, Mexico; 5 Regional Graduate Program in Biotechnology, Faculty of Chemical and Biological Sciences, Autonomous University of Sinaloa, Culiacan 80000, Sinaloa, Mexico; claudialeonsicairos@uas.edu.mx; 6 Unidad de Medicina Familiar No. 21, Mexican Social Security Institute, La Cruz de Elota 82700, Sinaloa, Mexico; tps48_@hotmail.com

**Keywords:** neonatal sepsis, early-onset sepsis, late-onset sepsis, antimicrobial resistance, biomarkers

## Abstract

The objective of this study was to determine the epidemiological, clinical, and laboratory characteristics of newborns with sepsis in northwestern Mexico, identify the microorganisms causing early- and late-onset sepsis, and assess antimicrobial resistance. Additionally, it sought to associate neonatal characteristics with antimicrobial resistance or mortality. A retrospective study was conducted from August 2021 to April 2023, during which 8382 neonatal clinical records were analyzed to collect epidemiological, clinical, and laboratory characteristics, as well as microorganisms isolated from neonates and their antimicrobial resistance profiles. Of these, 314 neonates with sepsis were included. The incidence of neonatal sepsis was 4% (314/8382), and the mortality was 12.7% (40/314); late-onset sepsis (65.3%) was more frequent than early-onset sepsis (34.7%). *Staphylococcus epidermidis* was the most frequently isolated bacterium in neonates with sepsis (both early- and late-onset). Gram-positive bacteria, such as *Staphylococcus hominis* and *Enterococcus faecium*, were associated with early-onset sepsis, whereas fungi, particularly *Candida albicans*, were associated with late-onset sepsis. Of the microorganisms, 52.6% were multidrug resistant (MDR), 10.8% were extensively drug resistant (XDR), and 5.5% were pan-drug resistant (PDR). Low birth weight, prematurity, cesarean section, mechanical ventilation, tachycardia, and low hemoglobin and platelet levels, among others, were associated with XDR or MDR microorganisms. In contrast, low birth weight, mechanical ventilation, stroke, unexpected delivery, respiratory distress, tachycardia, convulsive crisis, high procalcitonin, urea, and AST/TGO levels, among others, were associated with mortality. The incidence, types of sepsis, antimicrobial resistance, and associations identified in this study will aid in diagnosing neonatal sepsis earlier and may reduce mortality in our region.

## 1. Introduction

Neonatal sepsis is a clinical syndrome in newborns (28 days of age or less) characterized by systemic signs of infection and the isolation of a microorganism from the blood. Neonatal sepsis can lead to organ dysfunction in newborns due to a dysregulated host response to infection [1]. Neonatal sepsis is a significant public health problem worldwide, with an estimated incidence of 22 cases per 1000 neonates and a mortality of 11–19%; for Mexico, it has an incidence of 4.7 cases per 1000 neonates and a mortality of 4–15 per 1000 live births [2,3]. Neonatal sepsis is classified into two categories based on the time of diagnosis: early-onset and late-onset. The former is diagnosed within the first 72 h of life, and its etiology is related to neonatal infection during pregnancy. Neonates can acquire the microorganisms through the placenta or during passage through the birth canal [4,5].

Some microorganisms associated with early-onset sepsis, such as Group B Streptococcus or Escherichia coli, are responsible for most cases [6,7,8]. Late-onset sepsis is diagnosed after three days of life, and its transmission is associated with nosocomial and community-acquired infections [9]. Microorganisms include *Staphylococcus aureus*, coagulase-negative Staphylococci, *Staphylococcus epidermidis*, *Candida*, and others [10,11,12].

The prognosis of neonates with sepsis depends not only on the microorganisms that infect the neonate but also on the antimicrobial resistance of these pathogens. Antimicrobial resistance is a phenomenon that has been increasing worldwide over the years; in fact, antimicrobial resistance is considered one of the priority public health problems and a current pandemic [13]. The importance of antimicrobial resistance in neonates with sepsis lies in the high probability of treatment failure to eradicate microorganisms, leading to prolonged septic states or the need for potent antimicrobials; both situations can result in the development of underlying neonatal diseases or death [14]. Antibiotic resistance in bacteria that cause neonatal sepsis has increased over the years, particularly among group B streptococcus bacteria. Between 2006 and 2018, penicillin resistance increased from 1.6% to 54.5% [15,16], while *E. coli* increased ampicillin resistance from 76% to 92.8% [17]. Studies indicate that antibiotic resistance is a risk factor for mortality in newborns and that the mortality rate may increase from 12% to 37.5% in infections caused by multidrug-resistant (MDR) strains compared to non-MDR strains [18].

The identification or early diagnosis of sepsis in neonates is a crucial step in providing the best treatment to eradicate the microorganisms that infect neonates. Therefore, it is essential to identify the characteristics of neonates associated with sepsis, which can serve as a biomarker to aid in the early diagnosis of sepsis. Some studies that have associated epidemiologic, clinical, or laboratory data with antimicrobial resistance or sepsis provide further evidence for the existence of biomarkers or identify new candidates; however, more studies are needed [19,20,21,22].

The prevalence of neonatal sepsis in Mexico is high, even higher than in other countries; moreover, the focus of research on neonatal sepsis and the associations of neonatal characteristics with antimicrobial resistance of microorganisms or death in Mexico is null. Furthermore, biomarkers related to predicting mortality or antibiotic resistance in microorganisms that cause neonatal sepsis are scarce. Therefore, this study aimed to determine the epidemiological and clinical characteristics, as well as laboratory data, of neonates with sepsis in northwestern Mexico. Additionally, the microorganisms causing early- and late-onset sepsis, as well as the antimicrobial agents used, were identified. Finally, the characteristics of neonates with sepsis (epidemiologic, clinical, and laboratory data) were associated with antimicrobial resistance of microorganisms or neonatal mortality.

## 2. Methods

### 2.1. Study Subjects

This was a historical, analytical, and observational study with a retrospective approach, which included neonates with sepsis and positive blood cultures for microorganisms born at the Women’s Hospital of Sinaloa between August 2021 and April 2023. In the present work, newborns with signs and symptoms of sepsis, confirmed by positive blood cultures, were included. Those with negative blood cultures were excluded, and neonates without clinical records were also eliminated. Supplementary Figure S1 illustrates the distribution of neonates included in this study. Neonatal sepsis is defined as a clinical syndrome in newborns (28 days of age or less) characterized by systemic signs of infection and the isolation of pathogenic microorganisms from the bloodstream [1]. Early-onset sepsis is a clinical syndrome characterized by signs and symptoms that manifest within the first 72 h of life. In contrast, late-onset sepsis is defined by clinical signs and symptoms that begin after 72 h of life [4,23].

### 2.2. Data Collected on Neonates

Data on neonates with sepsis born at the Women’s Hospital were collected from medical records. Epidemiological characteristics of neonates with sepsis and their mothers were collected, including sex, birth weight, week of gestation, birth type, APGAR score at 1st minute of life, perinatal risk factors, day hospitalized, and mother age, among others. Clinical data of neonates were collected for the most prevalent symptoms. Finally, laboratory data include hemoglobin, hematocrit, total leukocytes, neutrophils, lymphocytes, monocytes, eosinophils, platelets, mean platelet volume, procalcitonin, bilirubin, creatinine, urea, ALT/TGP, and AST/TGO.

### 2.3. Identification of Microorganisms and Antimicrobial Resistance

Blood cultures were obtained from all neonates with signs and symptoms associated with sepsis, and microorganism identification and antimicrobial resistance was determined using the MicroScan WalkAway 96 automated system (Beckman Coulter, Brea, CA, USA), according to the manufacturer’s instructions and guidelines developed by the Clinical and Laboratory Standards Institute (CLSI) [24]. Only microorganisms isolated from neonates were considered; those contaminants in the blood cultures were discarded. Microorganisms were classified into two categories: bacteria (including Gram-positive and Gram-negative species) and fungi, with species identification provided for both groups. Regarding antimicrobial resistance, the antibiotics and antifungals tested were those recommended in the Mexican health system. The resistance of the microorganisms was classified as follows: (i) bacteria: those resistant to ≥three different categories of antibiotics were classified as multidrug resistant (MDR), those resistant to ≥6 different categories of antibiotics were classified as extensively drug resistant (XDR), and pan-drug-resistant bacteria were those resistant to all categories of antibiotics tested [25] and (ii) fungi: MDR were those resistant to more than one antifungal from two antifungal classes, while XDR were those resistant to more than one antifungal from three antifungal classes, and PDR were those resistant to all four antifungal classes [26,27].

### 2.4. Statistical Analysis

The Kolmogorov–Smirnov test was used to assess the normality of the samples. Measures of central tendency, including the mean, dispersion with standard deviation, and variance, were used to organize quantitative variables. Percentages and frequencies were used for qualitative variables. The X-squared test was used to compare qualitative variables, and binary or linear logistic regression was used in univariate and multivariate models to investigate the association between neonatal characteristics and antimicrobial resistance or mortality. A *p*-value < 0.05 was considered statistically significant. Student’s *t*-test or Mann–Whitney U-test was used for qualitative variables, depending on their distribution relative to the Gaussian curve. SPSS version 20 (IBM) was used for statistical analysis.

### 2.5. Ethical Consideration

This study was approved by the Ethics Committee of the Women’s Hospital, Ministry of Health, on 15 June 2022 (No. 202206-114). The principles of the Declaration of Helsinki of the World Medical Association were followed, with a waiver of consent generally granted due to the need to collect only routinely available clinical data, as no additional study-specific diagnostic testing was required.

## 3. Result

### 3.1. Characteristics of Neonates with Sepsis Included in This Study

Of the 8382 neonates born at the Women’s Hospital, the Secretariat of Health of Sinaloa, between 1 August 2021 and 30 April 2023, 314 with sepsis were included in this study, with an incidence of 4%; the distribution of neonates included and excluded from this study is shown in Supplementary Figure S1.

The epidemiologic, clinical, and laboratory characteristics of the neonates with sepsis are presented in Table 1. Among the epidemiologic characteristics of the neonates, 58% (182/314) were male, 41% (129/314) were female, and 1% (3/314) were of indeterminate sex. Regarding birth weight, 36% (115/314) weighed > 2500 g, similar proportions were found in neonates weighing 1500–2499 g and 1000–1499 g (21.3%, 67/317 and 22.3%, 70/314, respectively), and 19.7% (62/314) weighed < 999 g. Regarding gestational age, proportions ranging from 13.4% to 19.4% were found in neonates with 29 to >39 weeks, while the highest proportion was found in extremely preterm neonates with <28 weeks (22.3%, 70/314) (Table 1). Cesarean delivery was more frequent (57.3%, 180/317) than vaginal delivery (42.7%, 134/314). Regarding the APGAR score in the first minute of life, most neonates scored >7 (58.9%, 185/314), followed by 4 to 6 (30.9%, 91/314) and <3 (10.2%, 32/314).

In terms of perinatal risk, urinary tract infection was the most frequent (34.7%, 109/314), with similar proportions of unexpected delivery and chorioamnionitis (4.5%, 14/314, and 4.1%, 13/314, respectively) and lower proportions of respiratory infection (0.3%, 1/314) (Table 1). Most newborns spent 0 to 49 days in the hospital (54.4%, 171/314), followed by 50 to 99 days (25.4%, 80/314), 100 to 199 days (14.9%, 47/314), and >200 days (5.1%, 16/314). Regarding maternal age, the highest proportion was found in mothers aged 16 to 25 years (61.4%, 193/314), then >26 years (37.5%, 118/314), and <15 years (0.9%, 3/314). Neonates with late-onset sepsis were more frequent (65.3%, 205/314) than those with early-onset sepsis (34.7%, 109/314). Mechanical ventilation was used in 44.9% (129/314) of neonates, 15.7% (45/314) had a stroke, and 12.7% (40/314) of neonates died (Table 1).

Several symptoms were observed in neonates with sepsis, as shown in Table 1. Respiratory distress was the most frequent (71.3%, 224/314), followed by tachycardia (25.8%, 81/314), fever (20.4%, 64/314), reticulated or mottled coloration (19.4%, 61/314), jaundice (18.2%, 57/314), tachypnea (14.6%, 46/314), and capillary refill >2 s (14%, 44/314); other symptoms were observed in smaller proportions, such as apnea (8.3%, 26/314), pallor (8%, 25/314), low mean arterial pressure (7.6%, 24/314), cyanosis and hypothermia (6.4%, 20/314), and others less than 6% (bradycardia, abdominal distention, bradypnea, vomiting, and convulsions) (Table 1).

Other essential data collected from the neonates were laboratory data; Table 1 shows the means and standard deviations of these data. According to the normal values of the newborns’ laboratory data, some parameters of the newborns with sepsis were altered, such as hematocrit (40.3% ± 9.32), total leukocytes (12.36 × 10^3^ µL ± 6.29), procalcitonin (10.1 mg/dL ± 25.23), direct bilirubin (1.7 ng/dL ± 2.49), indirect bilirubin (1.8 mg/dL ± 3.05), creatinine (0.43 ± 2.49), ALT/TGP (59.9 ± 70.27), and AST/TGO (77.98 ± 124.13) (Table 1).

### 3.2. Microorganisms Isolated from Early- and Late-Onset Neonatal Sepsis

Among the 314 neonates with sepsis, several microorganisms were isolated as suspected culprits of neonatal infection. Overall, 77% (242/314) of microorganisms were bacteria (70% (171/242) Gram-positive and 30% (71/242) Gram-negative), while 23% (72/314) were fungi (Table 2). Regarding bacteria, 33 different species were found, with *Staphylococcus epidermidis* being the most frequent (25.2%, 61/242), followed by *Staphylococcus hominis* (10.7%, 26/242) and *Staphylococcus haemolyticus* (10.3%, 25/242), and, in smaller proportions, *Pseudomonas aeruginosa* (7%, 17/242), *Escherichia coli* (6.6%, 16/242), *Klebsiella pneumoniae*, and *Staphylococcus aureus* (6.1%, 15/242). The remaining bacteria had a proportion of less than 4.5% (Table 2). As for fungi, seven species were found, with *Candida albicans* (43%, 31/72) being the most frequent, followed by *Candida parapsilosis* (36.1%, 26/72) and *Candida guilliermondii* (7%, 5/23), and the remaining fungi had a proportion of less than 5.6% (Table 2).

Regarding the type of sepsis, 34.7% (109/314) belonged to early-onset sepsis, while 65.3% (205/314) belonged to late-onset sepsis (Table 3). Among all bacteria and fungi isolated, Table 3 lists the 15 most common bacteria and the five most frequent fungal species, ordered by the prevalence of late-onset sepsis. Overall, Gram-positive bacteria were more frequent in early-onset sepsis than in late-onset sepsis (81.6% vs. 40%, *p*: 0.0001); conversely, Gram-negative bacteria were more frequent in late-onset sepsis than in early-onset sepsis (25.8% vs. 16.5%), and fungi were more frequent in late-onset sepsis (34.1% vs. 1.83%, *p*: 0.0001) (Table 3). According to bacterial species, the most frequent bacterium in early-onset sepsis was *Staphylococcus epidermidis* (20.1%, 22/109), followed by *Staphylococcus hominis* (13.7%, 15/109), *Staphylococcus haemolyticus* (11%, 12/109), and *Staphylococcus aureus* (7.3%, 8/109), and the remaining bacteria had a proportion of less than 6.4%. In early-onset sepsis, only *Candida albicans* and *Candida glabrata* (0.9%, 1/109) were identified (Table 3). In late-onset sepsis, *Staphylococcus epidermidis* was also the most frequent (19%, 39/205), followed by *Pseudomonas aeruginosa* (8.3%, 17/205), *Staphylococcus haemolyticus* (6.3%, 13/205), *Staphylococcus hominis,* and *Klebsiella pneumoniae* (5.3%, 11/205), with the remaining bacteria accounting for less than 4.4% (Table 3). As for fungi, *Candida albicans* was the most frequent (14.6%, 30/205), followed by Candida parapsilosis (12.6%, 26/205), *Candida guilliermondii* (2.4%, 5/205), and the remaining fungi, which represented less than 1.9% (Table 3).

In addition, we found some bacteria associated with early-onset sepsis, such as *Staphylococcus hominis* (13.7% vs. 5.3%, *p*: 0.01) and *Enterococcus faecium* (3.6% vs. 0.5%, *p*: 0.032), while *Candida albicans* was associated with late-onset sepsis (14.6% vs. 0.9%, *p*: 0.0001) (Table 3).

### 3.3. Antimicrobial Resistance of Microorganisms Isolated from Neonates with Sepsis

Information on the antimicrobial resistance of microorganisms is presented in Table 4. A total of 287 microorganisms were analyzed; however, 27 did not have antibiograms, comprising 22 bacterial and 5 fungal species. In this analysis, 76.6% (220/287) were bacteria and 23.4% (67/287) were fungi. Overall, 87.4% (251/287) of microorganisms were resistant to any antibiotic, 52.6% (151/287) were multidrug resistant (MDR), 10.8% (31/287) were extensively drug resistant (XDR), and 5.5% (16/287) were pan-drug resistant (PDR). When comparing bacteria and fungi, the proportion of bacteria resistant to any antibiotic was higher than that of fungi (96.3% vs. 67.1%, *p*: 0.0001), a higher MDR incidence was found in bacteria than in fungi (65% vs. 11.9%, *p*: 0.0001); however, the proportion of MDR fungi was higher than that of bacteria (28.3% vs. 5.4, *p*: 0.0001), and the proportion of PDR was similar in bacteria and fungi (Table 4). Additionally, Supplementary Table S1 illustrates the antibiotic resistance among the most common Gram-positive, Gram-negative, and fungal pathogens identified in newborns. *S. epidermis* (32.5%), *S. hominis* (14.5%), and *S. haemolyticus* (13.2%) had the highest proportion of MDR, while *C. albicans* (35.4%), *C. parapsilosis* (19.3%), and *P. aeruginosa* (12.9%) had the highest proportion of XDR. The highest proportion of PDR was found in *S. epidermis* (25%), as well as in *P. aeruginosa* and *C. albicans* (12.5%, respectively).

According to the number of antimicrobials, 12.5% (36/287) were sensitive to all antimicrobials, 64.8% (186/287) were resistant to 1–8 antimicrobials, and 22.6% (65/287) were resistant to 9–16 antimicrobials. Among microorganisms, the proportion of fungi susceptible to all antimicrobials was higher than that of bacteria (32.8% vs. 6.3%, *p*: 0.0001); likewise, fungi were more resistant to two antimicrobials than bacteria (25.3% vs. 4.0%, *p*: 0.0001); while bacteria were more resistant to six antimicrobials (9.5% vs. 1.4%, *p*: 0.03); furthermore, only bacteria were resistant to 7–16 antimicrobials (47.2%), (Table 4). In addition, the resistance of microorganisms isolated from early- and late-onset sepsis was also analyzed (Supplementary Figure S2). The proportion of isolates susceptible to all antimicrobials and resistant to any antimicrobial was similar between early- and late-onset isolates (10.3% vs. 13.6% and 89.6% vs. 86.3%, respectively). Microorganisms isolated in early- onset were more often MDR than in late-onset (62.9% vs. 47.3%, *p*: 0.012), but microorganisms isolated in late-onset were more often XDR than in early-onset (14.7% vs. 3.1%, *p*: 0.002); the proportion of PDR was slightly higher in late-onset than in early-onset (7.4% vs. 2.1%) (Supplementary Figure S2).

### 3.4. Epidemiologic, Clinical, and Paraclinical Characteristics of Neonates with Sepsis Associated with Antimicrobial Resistance or Mortality

Associations were sought between characteristics of neonates with sepsis and antimicrobial resistance or mortality; microorganism resistance was divided into five groups: susceptible (12.5%, 36/287), RL3CA: resistant to less than three categories of antimicrobials (18.4%, 53/287), MDR (52.6%, 151/287), XDR (10.8%, 31/287), and PDR (5.5%, 16/287) (Supplementary Table S2). Some epidemiologic and clinical characteristics, as well as laboratory parameters, were associated with microorganisms exhibiting any of the above resistance categories or with mortality, as shown in Supplementary Table S2.

Binary or linear logistic regression was used to determine possible associations between neonatal characteristics and previous resistance-related sepsis or mortality. Table 5 shows all neonatal characteristics associated with some resistance of sepsis-causing microorganisms analyzed in the univariate model, among which epidemiological characteristics, such as the low birth weight of neonates 1000–1499 g, were associated with XDR (OR: 2.44, 95% CI: 1.13–5.40, *p*: 0.024), while <999 g was associated with XDR (OR: 4.62, 95% CI: 2.12–10.05, *p*: <0.001) and PDR (OR: 3.35, 95% CI: 1.19–9.42, *p*: 0.022); very preterm 29–30 weeks gestation with XDR microorganisms (OR: 2.96, 95% CI: 1.23–6.97, *p*: 0.014), extremely preterm <28 weeks gestation with PDR (OR: 3.75, 95% CI: 1.35–10.4, *p*: 0.011); caesarean section with PDR (OR: 11.73, 95% CI: 1.52–90.17, *p*: 0.018); APGAR score of 04–06 (OR: 10.71, 95% CI: 1.34–85.5, *p*: 0.025), and <3 (OR: 5.41, 95% CI: 1.71–17.04, *p*: 0.004) with PDR; mechanical ventilation with PDR (OR: 3.95, 95% CI: 1.24–12.55, *p*: 0.02); 100–199 days of hospitalization with XDR (OR: 4.85, 95% CI: 2.18–10.31, *p*: <0.001); and maternal age >26 years with XDR (OR: 2.45, 95% CI: 1.15–5.23, *p*: 0.02). Neonatal symptoms such as tachycardia (OR: 4.76, 95% CI: 1.67–13.58, *p*: 0.004) and low mean arterial pressure were associated with PDR (OR: 6.13, 95% CI: 1.97–20.14, *p*: 0.002). Neonatal laboratory data such as low hemoglobin (OR: 2.28; 95% CI: 1.08–3.47; *p*: <0.001), hematocrit (OR: 6.44; 95% CI: 3.03–9.86; *p*: <0. 001), platelets (OR: 1.37, 95% CI: 0.71–2.02, *p*: <0.001), and high AST/TGO levels (OR: 64.7, 95% CI: 13.3–116.3, *p*: 0.014) were associated with XDR (Table 5). The multivariate model was also applied to neonatal data; as a result, neonatal birth weights 1000–1499 g (OR: 5.81, 95% CI: 1.46–23.11, *p*: 0.012) and <999 g (OR: 9.65, 95% CI: 2.75–33.91, *p*: <0.001) were associated with XDR microorganisms (Table 5).

On the other hand, characteristics of neonates with sepsis were also associated with mortality, including epidemiologic traits such as low birth weight 1000–1499 g (OR: 2.58, 95% CI: 1.42–5.75, *p*: 0.003); mechanical ventilation (OR: 6.98, 95% CI: 3.10–15.70, *p*: <0.001); stroke (OR: 8.75, 95% CI: 4.21–18.18, *p*: <0.001); perinatal risk factor unexpected delivery (OR: 6.58, 95% CI: 2.18–19.87, *p*: 0.001); and maternal age >26 years (OR: 2.07, 95% CI: 1.07–3.99, *p*: 0.029) (Table 6). Neonatal symptoms were also associated with mortality, such as respiratory distress (OR: 3.48, 95% CI: 1.30–9.07, *p*: 0.013), tachycardia (OR: 1.86, 95% CI: 0.95–3.67, *p*: 0.07), capillary refill >2 s (OR: 3.18, 95% CI: 1.49–6.79, *p*: 0.003), low mean arterial pressure (OR: 2.82, 95% CI: 1.08–7.35, *p*: 0.033), and seizure (OR: 24.92, 95% CI: 2.71–228.8, *p*: 0.004) (Table 6). In addition, laboratory data of neonates with sepsis were also associated with mortality as low platelets (OR: 61.18, 95% CI: 1.41–108.1, *p*: 0.011), elevated levels of mean platelet volume (OR: 1.63, 95% CI: 1.17–2.11, *p*: <0.001), procalcitonin (OR: 19.94, 95% CI: 10.28–29.59, *p*: <0.001), total bilirubin (OR: 1.78, 95% CI: 0.28–3.28, *p*: 0.02), direct bilirubin (OR: 2.28, 95% CI: 1.41–3. 04, *p*: <0.001), urea (OR: 18.4, 95% CI: 7.94–28.86, *p*: 0.001), and AST/TGO (OR: 106.9, 95% CI: 63.2–150.7, *p*: <0.001) (Table 6). In addition, mechanical ventilation (OR: 6.05, 95% CI: 2.18–16.73, *p*: 0.001), stroke (OR: 4.0, 95% CI: 1.65–9.69, *p*: 0.002), unexpected delivery (OR: 16.96, 95% CI: 4.23–67.69, *p*: <0.001), and convulsive crisis (OR: 38.89, 95% CI: 2.66–586.54, *p*: 0.007) were associated with mortality in a multivariate model (Table 6).

## 4. Discussion

Neonatal sepsis is a global public health problem in both developed and developing countries. It is the leading cause of neonatal death. Unfortunately, despite all the research, treatments, and technologies that have been invested in this condition, the statistics regarding morbidity and mortality remain alarming. In this study, we found an incidence of neonatal sepsis of 4% and a mortality of 12.7%; Gram-positive bacteria, specifically Staphylococcus epidermidis, were the most frequently isolated bacteria from neonates with sepsis. Gram-positive bacteria, Staphylococcus hominis, and Enterococcus faecium were associated with early-onset sepsis, while fungi, particularly Candida albicans, were associated with late-onset sepsis. The MDR category was related to sepsis-causing bacteria and microorganisms isolated from early-onset sepsis, whereas XDR was associated with fungi and microorganisms isolated from late-onset sepsis. Epidemiologic (including low birth weight, prematurity, low APGAR score, mechanical ventilation, stroke, prolonged hospital stay, unexpected delivery, and maternal age), clinical (including respiratory distress, capillary refill tachycardia >2 s, and convulsive crisis), and paraclinical (including low hemoglobin, hematocrit, platelets, and high mean platelet volume, procalcitonin, total and direct bilirubin, urea, and AST/TGO) factors were associated with XDR or MDR microorganisms and/or neonatal mortality.

The incidence of neonatal sepsis may vary depending on the study area; however, the incidence found in this work was similar to that reported in other studies in Mexico, with rates of 4.3% in Mexico City, 3.7% in Nuevo León, and 4.7% in Guadalajara [28,29,30]. Compared to other countries, a lower incidence was found in the United States (1.8%) and a higher incidence in India (17%) [31,32]; moreover, neonatal sepsis was more prevalent in males, and this is in line with other studies [31,32], and this is because the responses of females mature concerning gestation age, while in males, the immune response is similar in pre-term and term; females have better monocyte activation than males, which can protect against infections [33]. LOS was more prevalent in this study, which is in line with other work in Mexico, where LOS was 11.2% and EOS was 0.5% [34]. In terms of mortality, the mortality found in this study was lower compared to the overall mortality of neonatal sepsis (13%) or that of countries such as India (15.7%) [35,36]; however, the mortality in this study was higher than that found in the Netherlands (4%) or the United Kingdom (0.8%) [37,38]. Many factors are associated with mortality in sepsis neonates; among the most important are the microorganisms that infect neonates and antimicrobial resistance.

Many bacteria were identified in this study, with Gram-positive bacteria being more prevalent than Gram-negative bacteria. This finding is consistent with other studies [39,40] and is associated with early-onset sepsis. Regarding bacterial species, *Staphylococcus epidermidis* was the most frequent in both types of sepsis. In early-onset, our results differ from those of other studies because the most frequently isolated pathogens in this type of sepsis in Mexico and other countries are typically Group B Streptococcus or *Escherichia coli* [6,34,41,42]. However, *Staphylococcus epidermidis* is consistent with another study as the most frequently isolated pathogen from late-onset sepsis [43,44].

*Staphylococcus epidermidis* is a coagulase-negative staphylococcus that has gained significant importance in this disease; these bacteria have become a predominant pathogen in neonatal sepsis, especially in preterm infants [23]. *Staphylococcus epidermidis* has primarily been associated with late-onset sepsis, as it is a bacterium that is part of the skin microbiota and can pose a threat to preterm infants when combined with suboptimal clinical practices by healthcare providers [45]. However, this bacterium has become more critical in cases of early-onset sepsis because many premature infants require respiratory or cardiac assist devices. These medical devices are a risk factor for Staphylococcus epidermidis infection in premature infants during the first hours of life [46].

On the other hand, other studies have identified *Staphylococcus hominis* or *Enterococcus faecium* as etiologic agents for neonatal sepsis; however, there are no reports associating these pathogens with early-onset sepsis as found in this work [47,48,49]. Regarding late-onset sepsis, most Candida species were found in this type of sepsis; however, only *Candida albicans* was associated with late-onset sepsis. This finding is consistent with other studies in various countries, including the United States, Korea, Germany, Taiwan, Kuwait, and southeastern Mexico, where *Candida* spp. was the most frequent fungus in late-onset sepsis [23]. In most reports of *Candida* spp. presence in neonates with sepsis, few studies identify the *Candida* subspecies, as in our work. Identifying the fungal and bacterial species in neonatal sepsis is crucial for providing the most effective treatment options for this affected population.

The prognosis of neonates with sepsis depends on the infecting microorganism and the antimicrobial-resistant microorganisms causing neonatal sepsis. In this study, bacteria causing neonatal sepsis were associated with the MDR category, while fungi were associated with XDR and PDR in similar proportions. The prevalence of MDR fungi and bacteria varies depending on the population in which neonatal sepsis is studied. Different studies have found the prevalence of MDR bacteria ranging from 38% to 100% [19,50,51]. These data are higher than what we found in our study. On the other hand, there are few studies on antimicrobial resistance in fungi isolated from neonates with sepsis. Most studies reported low fungal resistance in neonatal sepsis [52,53]. Regarding the type of sepsis, this study found higher resistance in microorganisms isolated from late-onset sepsis than early-onset sepsis, and these data are consistent with other studies [54,55,56]. The high antibiotic resistance found in this work has profound clinical implications, by reducing antibiotic options to treat neonatal sepsis in our region; this high resistance in bacteria and fungus isolated from neonates has a relation with the indiscriminate use of antibiotics in Mexico, not only in clinical practice but also in food, livestock, and veterinary practices [57,58,59].

The antibiotic resistance found in this work is alarming; 16 microorganisms were found to be PDR. PDR microorganisms leave neonates with sepsis with virtually no treatment options. High antimicrobial resistance is associated with the use of inappropriate empiric treatment and delays in antimicrobial administration when the etiologic agent is unknown [60]. In addition, high levels of antimicrobial resistance may affect the prognosis of neonates by increasing mortality, days of hospitalization, complications, or altering laboratory data. The epidemiologic, clinical, and paraclinical characteristics of this study were associated with antimicrobial resistance, primarily XDR and PDR. Other studies have also associated various neonatal characteristics with antimicrobial resistance. Yusef et al. (2015) associated neonatal clinical characteristics with sepsis, including prematurity, multiple gestation, previous antibiotic use, mechanical ventilation, and elevated C-reactive protein levels, with multidrug-resistant (MDR) microorganisms [21]. Tsai MH et al. (2014) associated complications of neonates with sepsis such as a high-frequency oscillatory ventilator, central venous catheter use, previous episode of bacteremia, renal disease, and prior antibiotic exposure (third-generation cephalosporin, vancomycin, carbapenem, and antifungals) with Gram-negative MDR bacteria as the etiologic agent of neonatal sepsis [22]. Solomon et al. (2021) found an association between neonatal factors, such as prematurity, low birth weight, and late-onset sepsis with multidrug-resistant (MDR) Gram-negative bacteria [51]. These data are consistent with our results; however, most studies have looked for associations with MDR bacteria, unlike our study, which examined associations between neonatal characteristics and all microorganisms (Gram-positive and Gram-negative bacteria and fungi) across all antimicrobial categories (from susceptible to PDR).

The characteristics of neonates with sepsis were associated with antimicrobial resistance and/or mortality, and our findings were consistent with those of other studies. Zavaleta H et al. (2025) associated neonatal infections with higher morbidity, longer hospitalization, and reduced survival [34]. Vigani AG et al. (2008) associated hematologic malignancy and urinary catheter use with increased gentamicin resistance of sepsis-causing *Enterococcus faecalis* in Brazilian neonates [61]. Dharma-Permana PB et al. (2024) associated extreme or very extreme prematurity, cesarean section, and lack of prenatal corticosteroid use with increased antibiotic resistance of sepsis-causing microorganisms [62]. Wei HM et al. (2015) associated neonatal characteristics with mortality, including prolonged stay in the neonatal intensive care unit, prolonged time with multidrug-resistant (MDR) infection, prolonged intubation, use of a ventilator, and multiple types of catheters, as well as low platelet and neutrophil counts [20]. Bandyopadhyay et al. (2018) associated prematurity, low birth weight, ventilator mechanics, feeding without breast milk, and early onset of sepsis with mortality in neonates with sepsis [19]. The results of this study will help determine the most effective treatment for newborns with sepsis and identify the most prevalent microorganisms that cause neonatal sepsis, as well as their antibiotic resistance in our region. This study will also investigate the associations between neonatal characteristics and antimicrobial resistance. This information may help diagnose newborns with sepsis as early as possible and provide them with the best treatment. The longer newborns suffer from sepsis, the greater their risk of death. Furthermore, identifying associations with mortality is crucial for developing new, particular predictive biomarkers to prevent death in these patients. More research on neonatal sepsis is needed to protect and help this vulnerable population.

This is the first comprehensive study showing the distribution and association of microorganisms with early- and late-onset sepsis in Mexico. In addition, no previous research has linked antimicrobial resistance categories (MDR, XDR, and PDR) to microorganisms causing neonatal sepsis or to microorganisms isolated from early- or late-onset sepsis. Finally, no evidence from studies associates epidemiologic, clinical, and paraclinical characteristics of neonates with sepsis with antimicrobial resistance categories (MDR, XDR, and PDR). The limitation of this study was the small sample size. The larger the sample size, the greater the likelihood of finding associations. Additionally, we did not investigate viruses as a potential etiologic agent of neonatal sepsis. Moreover, the retrospective design and absence of long-term outcomes.

## 5. Conclusions

This study provides evidence that the incidence of neonatal sepsis was 4% and the mortality was 12% in the state of Sinaloa. Gram-positive bacteria, especially *Staphylococcus epidermidis*, were the primary etiologic agents of neonatal sepsis. Additionally, *Staphylococcus hominis* and *Enterococcus faecium* were associated with early-onset sepsis, while *Candida albicans* were associated with late-onset sepsis. Increased levels of antimicrobial resistance were observed in bacteria and fungi isolated from patients with early- or late-onset sepsis. Neonates’ epidemiologic, clinical, and paraclinical characteristics were associated with antimicrobial resistance and neonatal mortality. The data found in this work provide evidence to help medical personnel in our region to make a more rapid diagnosis of neonatal sepsis and the possible microorganisms that can infect neonates; in addition, to pay close attention to those neonates with characteristics associated with mortality to provide them with the best treatment and prevent death in this vulnerable population. All risk factors identified in this study may point the way to more effective therapies for neonatal sepsis. However, further studies are needed to demonstrate their validity.

## Figures and Tables

**Table 1:** Epidemiological, clinical, and laboratory data of neonates with sepsis.

Epidemiological Traits	*n* = 314 (%)
Sex	
Male	182 (58.0)
Female	129 (41.0)
Non-determined	3 (1.0)
Birth weight (gr)	
>2500	115 (36.6)
1500–2499	67 (21.3)
1000–1499	70 (22.3)
<999	62 (19.7)
Week gestation (WG)	
Full-term >39	42 (13.4)
Early-term 37–38	53 (16.9)
Late preterm 34–36	61 (19.4)
Moderate preterm 31–33	45 (14.3)
Very preterm 29–30	43 (13.7)
Extremely preterm <28	70 (22.3)
Birth type	
Vaginal birth	134 (42.7)
Cesarean section	180 (57.3)
APGAR score 1st minute of life (points)	
>7	185 (58.9)
4 to 6	97 (30.9)
<3	32 (10.2)
Perinatal risk factors	
Urinary tract infection	109 (34.7)
Unexpected birth	14 (4.5)
Chorioamnionitis	13 (4.1)
Respiratory infection	1 (0.3)
Days hospitalized	
0–49	171 (54.4)
50–99	80 (25.4)
100–199	47 (14.9)
>200	16 (5.1)
Mother’s age (years)	
<15	3 (0.9)
16–25	193 (61.4)
>26	118 (37.5)
Sepsis classification	
Late-onset	205 (65.3)
Early-onset	109 (34.7)
Mechanic ventilation	129 (44.9)
Stroke	45 (15.7)
Mortality	40 (12.7)
Neonate symptoms	*n* = 314 (%)
Respiratory difficulty	224 (71.3)
Tachycardia	81 (25.8)
Fever	64 (20.4)
Reticulated/marble coloration	61 (19.4)
Jaundice	57 (18.2)
Tachypnea	46 (14.6)
Capillary refill > 2 s	44 (14.0)
Apnea	26 (8.3)
Pallor	25 (8.0)
Low average blood pressure	24 (7.6)
Cyanosis	20 (6.4)
Hypothermia	20 (6.4)
Bradycardia	18 (5.7)
Abdominal distension	16 (5.1)
Bradypnea	12 (3.8)
Vomit	5 (1.6)
Convulsive crisis	5 (1.6)
Neonate laboratory data	Mean (S.D.)
Hemoglobin (gr/dL)	13.73 (±3.24)
Hematocrit (%)	40.3 (±9.32)
Total leukocytes (×10^3^ µL)	12.36 (±6.29)
Neutrophils (×10^3^ µL)	6.22 (±4.83)
Lymphocytes (×10^3^ µL)	3.62 (±2.04)
Monocytes (×10^3^ µL)	1.67 (±1.3)
Eosinophils (×10^3^ µL)	0.35 (±0.33)
Platelets (×10^3^ µL)	192 (±148)
Mean platelet volume (fL)	10.8 (±9.3)
Procalcitonin (ng/dL)	10.1 (±25.23)
Total bilirubin (mg/dL)	3.98 (±4.11)
Direct bilirubin (mg/dL)	1.7 (±2.49)
Indirect bilirubin (mg/dL)	1.8 (±3.05)
Creatinine (mg/dL)	0.43 (±2.49)
Urea (mg/dL)	33.76 (±26.96)
ALT/TGP (U/L)	54.9 (±70.27)
AST/TGO (U/L)	77.98 (±124.13)

Abbreviations: g: grams, dL: deciliter, mg: milligrams, ng: nanograms, U: units, L: liters, %: percentage, µL: microliter, ALT: Alanine aminotransferase, and AST: Aspartate aminotransferase. Normal values of laboratory data: hemoglobin 15–24 g/dL, hematocrit: 45–61%, total leukocytes: 4.4–11.3 × 10^3^ µL, neutrophils: 6−23.5 × 10^3^ µL, lymphocytes: 2.5–10.5 × 10^3^ µL, eosinophils: <2 × 10^3^ µL, platelets: 150–450 × 10^3^ µL, mean platelet volume: 6–9.5 fL, procalcitonin: <0.5–2.4 ng/mL, total bilirubin: <10 mg/dL, direct bilirubin: 0–0.4 mg/dL, indirect bilirubin: 0.1–1.0 mg/dL, creatinine: 0.35–0.40 mg/dL, urea: 11–36 mg/dL, ALT/TGP: 11–54 U/L, and AST/TGO: 25–75 U/L.

**Table 2:** General distribution of microorganisms isolated from neonates with sepsis.

Bacteria	*n* = (%)
	*n* = 242 (77)
Gram-positive	171 (70)
Gram-negative	71 (30)
Bacteria species	
*Staphylococcus epidermidis*	61 (25.2)
*Staphylococcus hominis*	26 (10.7)
*Staphylococcus haemolyticus*	25 (10.3)
*Pseudomonas aeruginosa*	17 (7.0)
*Escherichia coli*	16 (6.6)
*Klebsiella pneumoniae*	15 (6.1)
*Staphylococcus aureus*	15 (6.1)
*Serratia marcescens*	11 (4.5)
*Kocuria kristinae*	7 (2.8)
*Coagulase-negative Staphylococci*	5 (2.0)
*Enterococcus faecium*	5 (2.0)
*Streptococcus agalactiae*	4 (1.6)
*Micrococcus luteus*	4 (1.6)
*Enterococcus faecalis*	4 (1.6)
*Staphylococcus lentus*	3 (1.2)
*Aerococcus viridans*	3 (1.2)
*Stenotrophomonas maltophilia*	2 (0.8)
*Burkholderia cepacia*	2 (0.8)
*Proteus mirabilis*	2 (0.8)
*Staphylococcus warneri*	2 (0.8)
*Staphylococcus lugdunensis*	1 (0.4)
*Enterobacter cloacae*	1 (0.4)
*Staphylococcus saprophyticus*	1 (0.4)
*Leuconostoc pseudomesenteroides*	1 (0.4)
*Acinetobacter haemolyticus*	1 (0.4)
*Elizabethkingia meningoseptica*	1 (0.4)
*Achromobacter xylosoxidans*	1 (0.4)
*Kytococcus sedentarius*	1 (0.4)
*Kocuria rosea*	1 (0.4)
*Citrobacter koseri*	1 (0.4)
*Staphylococcus capitis*	1 (0.4)
*Acinetobacter baumannii*	1 (0.4)
*Kocuria rhizophila*	1 (0.4)
Fungus	*n* = 72 (23)
*Candida albicans*	31 (43.0)
*Candida parapsilosis*	26 (36.1)
*Candida guilliermondii*	5 (7.0)
*Cryptococcus laurentii*	4 (5.6)
*Candida tropicalis*	3 (4.1)
*Candida glabrata*	2 (2.8)
*Candida ciferrii*	1 (1.4)
Total *n*: 314.	

**Table 3:** Bacteria and fungi that were identified in cases of early- and/or late-onset sepsis.

Microorganisms	Early-Onset	Late-Onset	*p*-Value
	*n* (%)	*n* (%)	
	*n* = 109 (34.7)	*n* = 205 (65.3)	
Gram-positive	89 (81.6) *	82 (40)	0.0001
Gram-negative	18 (16.5)	53 (25.8)	0.059
Fungus	2 (1.83)	70 (34.1) *	0.0001
Bacteria species			
*Staphylococcus epidermidis*	22 (20.1)	39 (19.0)	0.804
*Pseudomonas aeruginosa*	0 (0.0)	17 (8.3)	-
*Staphylococcus haemolyticus*	12 (11.0)	13 (6.3)	0.147
*Staphylococcus hominis*	15 (13.7) *	11 (5.3)	0.01
*Klebsiella pneumoniae*	4 (3.6)	11 (5.3)	0.502
*Escherichia coli*	7 (6.4)	9 (4.4)	0.435
*Serratia marcescens*	3 (2.7)	8 (3.9)	0.607
*Staphylococcus aureus*	8 (7.3)	7 (3.4)	0.120
*Kocuria kristinae*	3 (2.7)	4 (1.9)	0.638
*Coagulase-negative Staphylococci*	3 (2.7)	2 (1.0)	0.226
*Enterococcus faecalis*	2 (1.8)	2 (1.0)	0.511
*Enterococcus faecium*	4 (3.6) *	1 (0.5)	0.032
*Aerococcus viridans*	2 (1.8)	1 (0.5)	0.242
*Streptococcus agalactiae*	4 (3.6)	0 (0.0)	-
*Micrococcus luteus*	4 (3.6)	0 (0.0)	-
Others	14 (12.8)	10 (4.8)	-
Fungus			
*Candida albicans*	1 (0.9)	30 (14.6)	0.0001
*Candida parapsilosis*	0 (0.0)	26 (12.6)	-
*Candida guilliermondii*	0 (0.0)	5 (2.4)	-
*Cryptococcus laurentii*	0 (0.0)	4 (1.9)	-
*Candida tropicalis*	0 (0.0)	3 (1.4)	-
Others	1 (0.9)	2 (1.0)	-

The order of bacteria and fungi has been adjusted to their prevalence in late-onset sepsis. More than 33 species of bacteria and seven fungi were isolated; the 15 and 5 most prevalent are shown, respectively. From early-onset, other bacteria: *Staphylococcus lugdunensis*, *Burkholderia cepacia*, *Staphylococcus saprophyticus*, *Kytococcus sedentarius*, *Leuconostoc pseudomesenteroides*, *Proteus mirabilis*, *Enterobacter cloacae*, *Staphylococcus lentus*, *Kocuria rosea*, *Aerococcus viridans*, *Micrococcus luteus*, *Staphylococcus capitis*, *Acinetobacter baumanii*, and *Staphylococcus warneri*. Other fungi: *Candida glabrata*. From late-onset, other bacteria: *Burkholderia cepacia*, *Stenotrophomonas maltophilia*, *Elizabethkingia meningoseptica*, *Staphylococcus lentus*, *Aerococcus viridans*, *Proteus mirabilis*, *Citrobacter koseri*, *Staphylococcus warneri*, *Acinetobacter haemolyticus*, and *Kocuria rhizophila.* Other fungi: *Candida guilliermondii* and *Candida ciferrii*. Statistical significance was obtained using the chi-square test. *: ≤0.05 statistically significant.

**Table 4:** Antimicrobial resistance of bacteria and fungi isolated from neonates with sepsis.

Category	Total Microorganisms	Bacteria *n* = (%)	Fungi *n* = (%)	*p*-Value
	*n* = 287 (%)	*n* = 220 (76.6)	*n* = 67 (23.4)	
Resistant to any antimicrobial	251 (87.4)	206 (93.6) *	45 (67.1)	0.0001
MDR	151 (52.6)	143 (65.0) *	8 (11.9)	0.0001
XDR	31 (10.8)	12 (5.4)	19 (28.3) *	0.0001
PDR	16 (5.5)	13 (5.9)	3 (4.4)	0.654
Resistance to antimicrobials				
0	36 (12.5)	14 (6.3)	22 (32.8) *	0.0001
1	25 (8.7)	16 (7.2)	9 (13.4)	0.22
2	26 (9.0)	9 (4.0)	17 (25.3) *	0.0001
3	27 (9.4)	18 (8.1)	9 (13.4)	0.356
4	16 (5.5)	13 (5.9)	3 (4.4)	0.604
5	30 (10.4)	24 (10.9)	6 (8.9)	0.647
6	22 (7.6)	21 (9.5) *	1 (1.4)	0.03
7	20 (6.9)	20 (9.1)	-	-
8	20 (6.9)	20 (9.1)	-	-
9	15 (5.2)	15 (6.8)	-	-
10	17 (5.9)	17 (7.2)	-	-
11	6 (2.0)	6 (2.7)	-	-
12	13 (4.5)	13 (5.9)	-	-
13	4 (1.3)	4 (1.8)	-	-
14	5 (1.7)	5 (2.2)	-	-
15	3 (1.0)	3 (1.3)	-	-
16	2 (0.7)	2 (0.9)	-	-

Of the total samples (*n* = 314), 27 lacked an antibiogram, of which 22 were bacteria, and 5 were fungi, MDR: multidrug resistant, XDR: extremely resistant, and PDR: pan-drug resistant. The classification of resistance of bacteria was carried out based on the article by Magiorakos AP et al. [25] while the classification of fungus was by Arendrup & Patterson [26] and Jacobs et al. [27]. Statistical significance was obtained using the chi-square test. *: ≤0.05 statistically significant.

**Table 5:** Neonate characteristics associated with antimicrobial resistance of microorganisms.

**Univariate Model**				
**Neonate Characteristic**	**Type Resistance Associated**	**OR**	**95% CI**	***p*-Value**
**Birth Weight (gr)**				
1000–1499	XDR	2.44	1.13–5.40	0.024
<999	XDR	4.62	2.12–10.05	<0.001
	PDR	3.35	1.19–9.42	0.022
**Week gestation (WG)**				
Very preterm 29–30	XDR	2.96	1.23–6.97	0.014
Extremely preterm <28	PDR	3.75	1.35–10.4	0.011
**Birth type**				
Cesarean section	PDR	11.73	1.52–90.17	0.018
**APGAR score 1st minute life (points)**				
4 to 6	PDR	10.71	1.34–85.50	0.025
<3	PDR	5.41	1.71–17.04	0.004
**Mechanic ventilation**	PDR	3.95	1.24–12.55	0.02
**Days hospitalized**				
100–199	XDR	4.85	2.18–10.31	<0.001
**Mother’s age (years)**				
>26	XDR	2.45	1.15–5.23	0.02
**Neonate symptoms**				
Tachycardia	PDR	4.76	1.67–13.58	0.004
Low average blood pressure	PDR	6.13	1.97–20.14	0.002
**Neonate laboratory data**				
Low hemoglobin	XDR	2.28	1.08–3.47	<0.001
Low hematocrit	XDR	6.44	3.03–9.86	<0.001
Low platelets	XDR	1.37	0.71–2.02	<0.001
High AST/TGO	XDR	64.7	13.3–116.2	0.014
**Multivariate model**				
**Neonate characteristics**	**Type resistance associated**	**OR**	**95% CI**	***p*-Value**
**Birth weight (gr)**				
1000–1499	XDR	5.81	1.46–23.11	0.012
<999	XDR	9.65	2.75–33.91	<0.001

Abbreviation: OR: odds ratio; CI: confidence interval. For epidemiologic traits and neonate symptoms, binary logistic regression was used, while for laboratory data, linear logistic regression was used to obtain associations. Only the statistically significant characteristics of the neonates are shown.

**Table 6:** Neonate characteristics associated with mortality.

**Univariate Model**			
**Neonate Characteristic**	**OR**	**95% CI**	***p*-Value**
**Epidemiological traits**			
**Birth weight (gr)**			
1000–1499	2.58	1.42–5.75	0.003
**Mechanic ventilation**	6.98	3.10–15.70	<0.001
**Stroke**	8.75	4.21–18.18	<0.001
**Perinatal risk factors**			
Unexpected birth	6.58	2.18–19.87	0.001
**Mother’s age (years)**			
>26	2.07	1.07–3.99	0.29
**Neonate symptoms**			
Respiratory difficulty	3.48	1.30–9.07	0.013
Tachycardia	1.86	0.95–3.67	0.07
Capillary refill >2 s	3.18	1.49–6.79	0.003
Low average blood pressure	2.82	1.08–7.35	0.033
Convulsive crisis	24.92	2.71–228.8	0.004
**Neonate laboratory data**			
Low platelets	61.18	14.1–108.1	0.011
High mean platelet volume	1.63	1.17–2.11	<0.001
High procalcitonin	19.94	10.28–29.59	<0.001
High total bilirubin	1.78	0.28–3.28	0.02
High direct bilirubin	2.28	1.41–3.04	<0.001
High urea	18.4	7.94–28.86	0.001
High AST/TGO	106.9	63.2–150.7	<0.001
**Multivariate model**			
**Neonate characteristic**	**OR**	**95% CI**	***p*-Value**
Mechanic ventilation	6.05	2.18–16.73	0.001
Stroke	4.0	1.65–9.69	0.002
Unexpected birth	16.96	4.23–67.96	<0.001
Convulsive crisis	38.89	2.66–568.54	0.007

Abbreviation: OR: odds ratio; CI: confidence interval. For epidemiologic traits and neonate symptoms, binary logistic regression was used, while for laboratory data, linear logistic regression was used to obtain associations. Only the statistically significant characteristics of the neonates are shown.

## Data Availability

Data is contained within the article or Supplementary Materials. The original contributions presented in this study are included in this article/Supplementary Materials. Further inquiries can be directed to the corresponding authors.

## Supplementary Materials

The following supporting information can be downloaded at: https://www.mdpi.com/article/10.3390/pathogens14060588/s1, Figure S1: Distribution of neonates included in this study; Figure S2: Antimicrobial resistance of microorganisms by type of sepsis; Table S1: Distribution of antibiotic resistance by microorganism species; Table S2: Epidemiological and clinical traits, and laboratory data of neonates with sepsis related to antimicrobial resistance, and mortality.
