# Supplementary material for: Characteristics of Neonates with Sepsis Associated with Antimicrobial Resistance and Mortality in a Tertiary Hospital in Mexico: A Retrospective Observational Study
Source: Pathogens. 2025 Jun 14;14(6):588. doi: 10.3390/pathogens14060588 (PMC12195758; doi:10.3390/pathogens14060588)
Supplement: Supplementary file 1 [file pathogens-14-00588-s001.zip › Supplementary Table S1.pdf]

Supplementary Table S1. Distribution of antibiotic resistance by microorganism species

Bacteria species	MDR	XDR	PDR
**Gram-positive**	**n= 151 (%)**	**n= 31 (%)**	**n= 16 (%)**
Staphylococcus epidermidis	49 (32.5)	2 (6.4)	4 (25.0)
Staphylococcus hominis	22 (14.5)	0 (0.0)	0 (0.0)
Staphylococcus haemolyticus	20 (13.2)	0 (0.0)	1 (6.2)
Staphylococcus aureus	10 (6.6)	0 (0.0)	0 (0.0)
Kocuria kristinae	0 (0.0)	0 (0.0)	0 (0.0)
Coagulase-negative Staphylococci	5 (3.3)	0 (0.0)	0 (0.0)
Enterococcus faecium	1 (0.6)	2 (6.4)	0 (0.0)
Streptococcus agalactiae	0 (0.0)	0 (0.0)	0 (0.0)
Micrococcus luteus	0 (0.0)	0 (0.0)	0 (0.0)
Enterococcus faecalis	1 (0.6)	0 (0.0)	1 (6.2)
**Gram-negative**			
Pseudomonas aeruginosa	2 (1.3)	4 (12.9)	2 (12.5)
Escherichia coli	8 (5.2)	2 (6.4)	0 (0.0)
Klebsiella pneumoniae	7 (4.6)	3 (9.6)	1 (6.25)
Serratia marcescens	8 (5.2)	0 (0.0)	0 (0.0)
Stenotrophomonas maltophilia	0 (0.0)	0 (0.0)	0 (0.0)
Burkholderia cepacia	0 (0.0)	0 (0.0)	0 (0.0)
Proteus mirabilis	1 (0.6)	0 (0.0)	0 (0.0)
Enterobacter cloacae	1 (0.6)	0 (0.0)	0 (0.0)
Acinetobacter haemolyticus	0 (0.0)	0 (0.0)	0 (0.0)
Elizabethkingia meningoseptica	0 (0.0)	0 (0.0)	0 (0.0)
**Fungus**			
Candida albicans	4 (2.6)	11 (35.4)	2 (12.5)
Candida parapsilosis	3 (1.9)	6 (19.3)	1 (6.25)
Candida guilliermondii	1 (0.6)	1 (3.2)	0 (0.0)
Cryptococcus laurentii	0 (0.0)	0 (0.0)	0 (0.0)
Candida tropicalis	0 (0.0)	0 (0.0)	0 (0.0)
Candida glabrata	1 (0.6)	0 (0.0)	0 (0.0)
Candida ciferrii	0 (0.0)	0 (0.0)	0 (0.0)

This table shows the resistance to antibiotics by species of microorganism isolated from neonates with sepsis. MDR: multidrug-resistant, XDR: extremely resistant, and PDR: pandrug-resistant. The classification of bacterial resistance was based on the article by Magiorakos AP et al. (2012), while the classification of fungal resistance was by Arendrup & Patterson (2017) and Jacobs et al. (2022).
