# Supplementary material for: Characteristics of Neonates with Sepsis Associated with Antimicrobial Resistance and Mortality in a Tertiary Hospital in Mexico: A Retrospective Observational Study
Source: Pathogens. 2025 Jun 14;14(6):588. doi: 10.3390/pathogens14060588 (PMC12195758; doi:10.3390/pathogens14060588)
Supplement: Supplementary file 1 [file pathogens-14-00588-s001.zip › Supplementary Table S2.pdf]

Supplementary Table S2. Epidemiological and clinical traits, as well as laboratory data, of neonates with sepsis related to antimicrobial resistance and mortality.

Epidemiological traits	Total n (%) n=287	Antimicrobial resistance<br>Susceptible n (%) n= 36 (12.5)	RL3AC n (%) n= 53 (18.4)	MDR n (%) n= 151 (52.6)	XDR n (%) n= 31(10.8)	PDR n (%) n=16 (5.5)	*p* value	Mortality<br>Alive n (%) n= 244 (85.1)	Dead n (%) n= 43 (14.9)	*p* value
**Birth weight (gr)**										
> 2500	105 (36.6)	9 (25.0)	20 (37.7)	72 (47.7)*	2 (6.5)	2 (12.5)	<0.001	100 (41.0)*	5 (11.6)	<0.001
1500-2499	59 (20.6)	7 (19.4)	11 (20.8)	36 (23.8)	2 (6.5)	3 (18.8	0.388	49 (20.1)	10 (23.3)	0.635
1000- 1499	65 (22.6)	15 (41.7	13 (24.5)	21 (13.9)	12 (38.7)*	4 (25.0)	0.007	42 (17.2)	12 (27.9)	0.372
< 999	58 (20.2)	5 (13.9)	9 (17.0)	22 (14.6)	15 (48.4)*	7 (43.8)*	<0.001	42 (17.2)	16 (37.2)*	0.003
**Week of gestation (WG)**										
Full-term >39	57 (19.8)	5 (13.9)	9 (17.0)	41 (27.2)	2 (6.5)	2 (12.5)	0.112	56 (23.0)*	3 (7.0)	0.017
Early-term 37-38	26 (9.1)	2 (5.6)	4 (7.5)	19 (12.6)	1 (3.2)	0 (0.0)	0.228	22 (9.0)	4 (9.3)	0.952
Late preterm 34- 36	58 (20.2)	4 (11.1)	15 (28.3)*	35 (23.2)	1 (3.2)	2 (12.5)	0.042	51 (20.9)	6 (14.0)	0.292
Moderate preterm 31-33	40 (13.9)	7 (19.4)	8 (15.1)	13 (8.6)	4 (12.9)	1 (6.3)	0.717	31 (12.7)	9 (20.9)	0.151
Very preterm 29-30	40 (13.9)	9 (25.0)	6 (11.3)	23 (15.2)	9 (29.0)*	3 (18.3)	0.032	33 (13.5)	7 (16.3)	0.631
Extremely preterm <28	65 (22.6)	9 (25.0)	11 (20.8)	23 (15.2)	14 (45.2)*	8 (50.0)*	<0.001	51 (20.9)	14 (32.6)	0.092
**Sex**										
Male	125 (43.6)	18 (50.0)	23 (43.4)	65 (43.0)	10 (32.2)	9 (56.3)		107 (43.9)	18 (41.9)	
Female	159 (55.4)	14 (47.2)	30 (56.6)	84 (55.6)	21 (67.7)	7 (43.8)	0.468	135 (55.3)	14 (55.8)	0.661
Non determined	3 (1.0)	1 (2.8)	0 (0.0)	2 (1.3)	0 (0.0)	0 (0.0)		2 (0.8)	1 (2.3)	
**Birth type**										
Vaginal birth	120 (41.8)	20 (55.6)	22 (41.5)	65 (43.0)	12 (38.7)	1 (6.3)	0.017	100 (41.0)	20 (46.5)	0.498
Caesarean section	167 (58.2)	16 (44.4)	31 (58.5)	86 (57.0)	19 (61.3)	15 (93.8)*		144 (59.0)	23 (53.5)	
**APGAR score 1st minute of life (points)**										

> 7	167 (58.2)	18 (50.0)	37 (69.8)*	96 (63.5)	15 (48.4)	1 (6.3)	0.010	144 (59.0)	23 (53.5)	0.885
04 to 06	94 (32.8)	16 (44.4)	14 (26.4)	42 (27.8)	12 (38.7)	10 (62.5)*	0.044	78 (32.0)	16 (37.2)	0.763
< 3	26 (9.1)	2 (5.6)	2 (3.8)	13 (8.6)	4 (12.9)	5 (31.3)*	0.012	22 (9.0)	4 (9.3)	0.952
**Mechanic ventilation**	129 (44.9)	18 (50.0)	22 (41.7)	60 (39.7)	17 (54.8)	12 (75.0)*	0.046	94 (38.5)	35 (81.4)*	<0.001
**Stroke**	45 (15.7)	8 (22.2)	7 (13.2)	22 (14.6)	4 (12.9)	4 (25.0)	0.567	24 (9.8)	21 (48.8)*	<0.001
**Perinatal risk factors**										
Rupture of membranes	5 (1.7)	1 (2.8)	0 (0.0)	3 (2.0)	0 (0.0)	1 (6.3)	0.794	3 (1.2)	2 (4.7)	0.149
Chorioamnionitis	10 (3.5)	1 (2.8)	3 (1.9)	5 (3.3)	0 (0.0)	1 (6.3)	0.793	10 (4.1)	0 (0.0)	0.177
Urinary tract infection	92 (32.1)	17 (47.2)	18 (34.0)	46 (30.5)	10 (32.3)	1 (6.3)	0.099	84 (34.4)	8 (18.6)	0.040
Respiratory infection	1 (0.3)	0 (0.0)	0 (0.0)	1 (0.7)	0 (0.0)	0 (0.0)	0.928	1 (0.4)	0 (0.0)	-
Unexpected birth	14 (4.9)	2 (5.6)	1 (1.9)	7 (4.6)	4 (12.9)	0 (0.0)	0.134	1 (2.9)	7 (16.3)*	<0.001
**Days hospitalized**										
0-49	156 (54.4)	15 (41.7)	27 (50.9)	104 (68.9)*	5 (16.1)	5 (31.3)	<0.001	129 (52.9)	24 (62.8)	0.28
50-99	72 (25.4)	14 (38.9)	14 (26.4)	27 (17.9)	11 (35.5)	7 (43.8)	0.077	65 (26.2)	9 (20.9)	0.462
100-199	46 (16.0)	7 (19.4)	10 (18.9)	14 (9.3)	13 (41.9)*	2 (12.5)	<0.001	39 (16.0)	7 (16.3)	0.961
> 200	13 (4.5)	0 (0.0)	2 (3.8)	6 (9.3)	2 (6.5)	2 (12.5)	0.268	12 (4.9)	0 (0.0)	-
**Mother's age (years)**										
< 15	4 (1.3)	1 (2.7)	1 (1.8)	2 (1.3)	0 (0.0)	0 (0.0)	0.779	2 (0.8)	0 (0.0)	-
16 - 25	173 (60.3)	28 (77.8)	34 (64.2)	86 (57.0)	14 (45.2)	11 (6.8)	0.104	154 (63.1)*	19 (44.2)	0.019
> 26	110 (38.3)	7 (19.4)	18 (34.0)	62 (41.1)	18 (58.1)*	5 (31.3)	0.049	87 (35.7)	23 (53.5)*	0.026
**Gestation number**										
1	98 (34.1)	16 (44.4)	19.0 (35.8)	46 (30.6)	14 (45.2)	3 (18.8)	0.184	86 (35.2)	12 (27.9)	0.376
> 2	189 (65.8)	20 (55.6)	34 (64.2)	105 (69.5)	17 (54.8)	13 (81.2)	0.220	158 (64.7)	31 (72.0)	0.252
**Sepsis classification**										
Early onset	97 (33.8)	10 (27.8)	21 (39.6)	61 (40.4)	3 (9.7)	2 (12.5)	0.184	88 (36.1)	9 (20.9)	0.050
Late onset	190 (66.2)	26 (72.2)	32 (60.4)	90 (59.6)	28 (90.3)	14 (87.5)		156 (63.9)	34 (79.1)*	
**Mortality**	43 (15.0)	4 (11.1)	9 (17.0)	21 (13.9)	7 (22.6)	2 (12.5)	0.435	-	-	
**Neonate´s symptoms**	**Total**	**Susceptible**	**RL3AC**	**MDR**	**XDR**	**PDR**	***p***	**Alive**	**Dead**	***p***
Respiratory difficulty	205 (71.4)	33 (91.6)	17 (32.0)	103 (68.2)	23 (74.9)	14 (87.5)	0.426	167 (68.4)	38 (88.7)*	0.028
Tachycardia	78 (27.2)	10 (27.8)	11 (20.8)	37 (24.5)	10 (32.3)	10 (62.5)*	0.016	63 (25.8)	15 (34.9)*	0.048
Fever	62 (21.6)	4 (11.1)	14 (26.4)	32 (21.2)	6 (19.4)	6 (37.5)*	0.035	52 (21.3)	10 (23.3)	0.054
Reticulated/marble coloration	65 (22.6)	8 (22.2)	15 (28.3)	32 (21.2)	6 (19.4)	4 (25.0)	0.505	52 (21.3)	13 (30.2)	0.197
Jaundice	51 (17.8)	5 (13.9)	8 (15.1)	33 (21.9)	3 (9.7)	2 (12.5)	0.304	43 (17.6)	8 (18.6)	0.906

Tachypnea	42 (14.6)	5 (3.9)	9 (14.0)	21 (13.9)	4 (12.9)	3 (18.8)	0.983	40 (16.4)	2 (4.7)	0.070
Capillary refill > 2 seconds	42 (14.6)	10 (27.8)	7 (13.2)	19 (12.6)	5 (16.1)	1 (6.3)	0.072	16 (6.6)	13 (30.2)*	0.002
Apnea	22 (7.7)	2 (5.6)	3 (5.7)	13 (8.6)	1 (3.2)	3 (18.8)	0.379	19 (7.8)	3 (7.0)	0.854
Pallor	27 (9.4)	7 (19.4)	5 (9.4)	9 (6.0)	4 (12.9)	2 (12.5)	0.085	25 (10.2)	2 (4.7)	0.247
Low average blood pressure	23 (8.0)	2 (5.6)	2 (3.8)	5 (3.3)	2 (6.5)	5 (31.3)*	0.008	16 (6.6)	7 (16.3)*	0.027
Cyanosis	11 (3.8)	0 (0.0)	1 (1.9)	8 (5.3)	2 (6.5)	0 (0.0)	0.739	8 (3.3)	3 (7.0)	0.090
Hypothermia	20 (7.0)	1 (2.8)	5 (9.4)	8 (5.3)	4 (12.9)	2 (12.5)	0.604	17 (7.0)	3 (7.0)	0.765
Bradycardia	17 (5.9)	3 (8.3)	2 (3.8)	10 (6.6)	2 (6.5)	0 (0.0)	0.818	11 (4.5)	6 (14.0)	0.248
Abdominal distension	15 (5.2)	5 (13.9)	3 (5.7)	5 (3.3)	0 (0.0)	2 (12.5)	0.069	14 (5.7)	1 (2.3)	0.349
Bradypnea	12 (4.2)	1 (2.8)	0 (0.0)	8 (5.3)	1 (3.2)	2 (12.5)	0.226	8 (3.3)	4 (9.0)	0.070
Vomit	4 (1.4)	1 (2.8)	1 (1.9)	1 (0.7)	0 (0.0)	1 (6.3)	0.376	3 (1.2)	1 (2.0)	0.572
Convulsive crisis	4 (1.4)	0 (0.0)	1 (1.9)	2 (1.3)	1 (3.2)	0 (0.0)	0.556	1 (0.4)	4 (9.3)*	<0.001
**Neonate´s laboratory data**	**Total**	**Susceptible**	**RL3AC**	**MDR**	**XDR**	**PDR**	***p***	**Alive**	**Dead**	***p***
Hemoglobin (gr/dl)	13.7 (±3.5)	13.4 (±3.3)	13.7 (±3.4)	14.4 (±3.2)	11.7 (±2.3)*	12.8 (±2.0)*	0.002	13.8 (±3.2)	13.2 (±3.3)	0.287
Hematocrit (%)	40.4 (±9.3)	39.2 (±9.4)	40.3 (±9.7)	42.2 (±9.4)	34.7 (±6.6)*	38.0 (±5.9)	0.003	40.6 (±9.2)	39.3 (±9.4)	0.441
Total leukocytes (x10^3 µl)	12.5 (±6.3)	10.8 (±6.2)	11.8 (±5.3)	13.4 (±6.3)	11.6 (±6.3)	11.2 (±8.8)	0.073	12.7 (±6.3)	11.3 (±6.2)	0.107
Neutrophils (x10^3 µl)	6.3 (±4.8)	5.7 (±4.8)	6.1 (±4.2)	6.8 (±4.8)	5.4 (±4.5)	5.75 (±6.4)	0.222	6.4 (±4.8)	5.6 (±4.5)	0.275
Lymphocytes (x10^3 µl)	3.6 (±2.0)	3.1 (±1.7)	3.3 (±1.6)	3.9 (±2.1)	3.2 (±1.9)	3.3 (±3.1)*	0.007	3.7 (±2.0)	3.0 (±2.1)*	0.021
Monocytes (x10^3 µl)	1.6 (±1.2)	1.2 (±0.9)	1.5 (±1.0)	1.7 (±1.2)	2.1 (±1.6)	1.4 (±1.5)*	0.047	1.6 (±1.1)	1.7 (±1.6)	0.660
Eosinophils (x10^3 µl)	0.35 (±0.34)	0.25 (±0.23)	0.37 (±0.3)	0.3 (±0.3)	0.2 (±0.2)	0.3 (±0.2)	0.138	0.3 (±0.3)	0.2 (±0.3)*	0.041
Platelets (x10^3 µl)	191.5 (±145)	165.5 (±145.1)	184.9 (±124.5)	218.8 (±153.2)	132.0 (±130.1)*	135.1 (±115.1)*	0.011	200 (±148.5)	139.6 (±116.0)*	0.007
Mean platelet volume (fL)	10.8 (±1.1)	10.5 (±0.87)	10.8 (±1.1)	10.7 (±1.1)	10.9 (±1.2)	11.7 (±1.3)	0.111	10.6 (±1.0)	12.3 (±1.2)*	<0.001
Procalcitonin (ng/dL)	10.8 (±26.0)	7.9 (±21.0)	11.6 (±28.1)	10.6 (22.4)	9.3 (±23.0)	20.1 (±54.2)	0.881	7.8 (±18.3)	27.7 (±47.7)*	0.032
Total bilirubin (mg/dL)	3.9 (±4.1)	3.6 (±3.5)	3.2 (±3.3)	4.5 (±4.5)	3.9 (±4.1)	1.3 (±1.09)	0.170	3.6 (±4.1)	5.4 (±3.7)*	0.002
Direct bilirubin (mg/dL)	1.6 (±2.3)	2.2 (±2.7)	1.8 (±2.2)	2.5 (±3.8)	1.4 (±2.0)	0.5 (±0.3)*	0.025	1.7 (±3.2)	2.0 (±2.4)	<0.001
Indirect bilirubin (mg/dL)	1.8 (±3.0)	1.3 (±1.9)	1.0 (±1.8)	1.2 (±1.8)	2.6 (±3.6)	0.8 (±1.7)	0.072	1.2 (±1.8)	3.4 (±3.2)*	0.023
Creatinine (mg/dL)	0.4 (±0.3)	0.4 (0.2)	0.4 (±0.3)	0.4 (±0.2)	0.5 (±0.5)	0.3 (±1.3)	0.832	0.4 (±0.2)	0.6 (±0.5)*	0.008
Urea (mg/dL)	33.9 (±27.3)	39.2 (±24.8)	39.9 (±27.2)	24.8 (±20.0)	39.4 (±46.6)	42.8 (±15.2)	0.342	30.7 (±21.9)	49.1 (±41.9)*	<0.001

ALT/TGP (U/L)	54.9 (±71.8)	60.4 (±65.5)	78.2 (±106.7)	36.9 (±39.9)	72.9 (±95.6)	72.2 (±60.6)	0.075	48.8 (±65.4)	76.3 (±98.2)	0.415
AST / TGO (U/L)	77.5 (±125)	92.6 (±158.2)	74.6 (±79.4)	49.7 (±44.7)	132.1 (±166.6)*	54.9 (±23.2)	0.004	55.2 (±48.1)	162.2 (±243.8)*	0.001

Of the total samples (n = 314), 27 lacked an antibiogram; of these, 22 were bacterial and five were fungal. Abbreviations: g: grams, dL: deciliter, mg: milligrams, ng, nanograms, U: units, L: liters, %: percentage, µl: microliter ALT: Alanine aminotransferase, AST: Aspartate aminotransferase, RL3CA: resistant to less than three antimicrobial categories, MDR: multidrug resistant, XDR: extremely resistant, PDR: pandrug resistant. Normal values of laboratory data: hemoglobin 15-24 g/dL, hematocrit: 45-61%, total leukocytes: 4.4-11.3x10^3 µl, neutrophils: 6 -23.5 x10^3 µl, lymphocytes: 2.5-10.5 x10^3 µl, eosinophils: <2 x10^3 µl, platelets: 150-450 x10^3 µl, mean platelet volume: 6-9.5 fL, procalcitonin: <0.5-2.4 ng/ml, total bilirubin: <10 mg/dl, direct bilirubin: 0- 0.4 mg/dl, indirect bilirubin: 0.1 - 1.0 mg/dL, creatinine: 0.35-0.40 mg/dl, urea: 11-36 mg/dl, ALT/TGP: 11-54 U/L, and AST / TGO: 25-75 U/L. Kruskal-Wallis and Mann-Whitney tests were applied to get statistical significance. *: ≤0.05.

.
