# Supplementary figures and images for: Characteristics of Neonates with Sepsis Associated with Antimicrobial Resistance and Mortality in a Tertiary Hospital in Mexico: A Retrospective Observational Study
Source: Pathogens. 2025 Jun 14;14(6):588. doi: 10.3390/pathogens14060588 (PMC12195758; doi:10.3390/pathogens14060588)

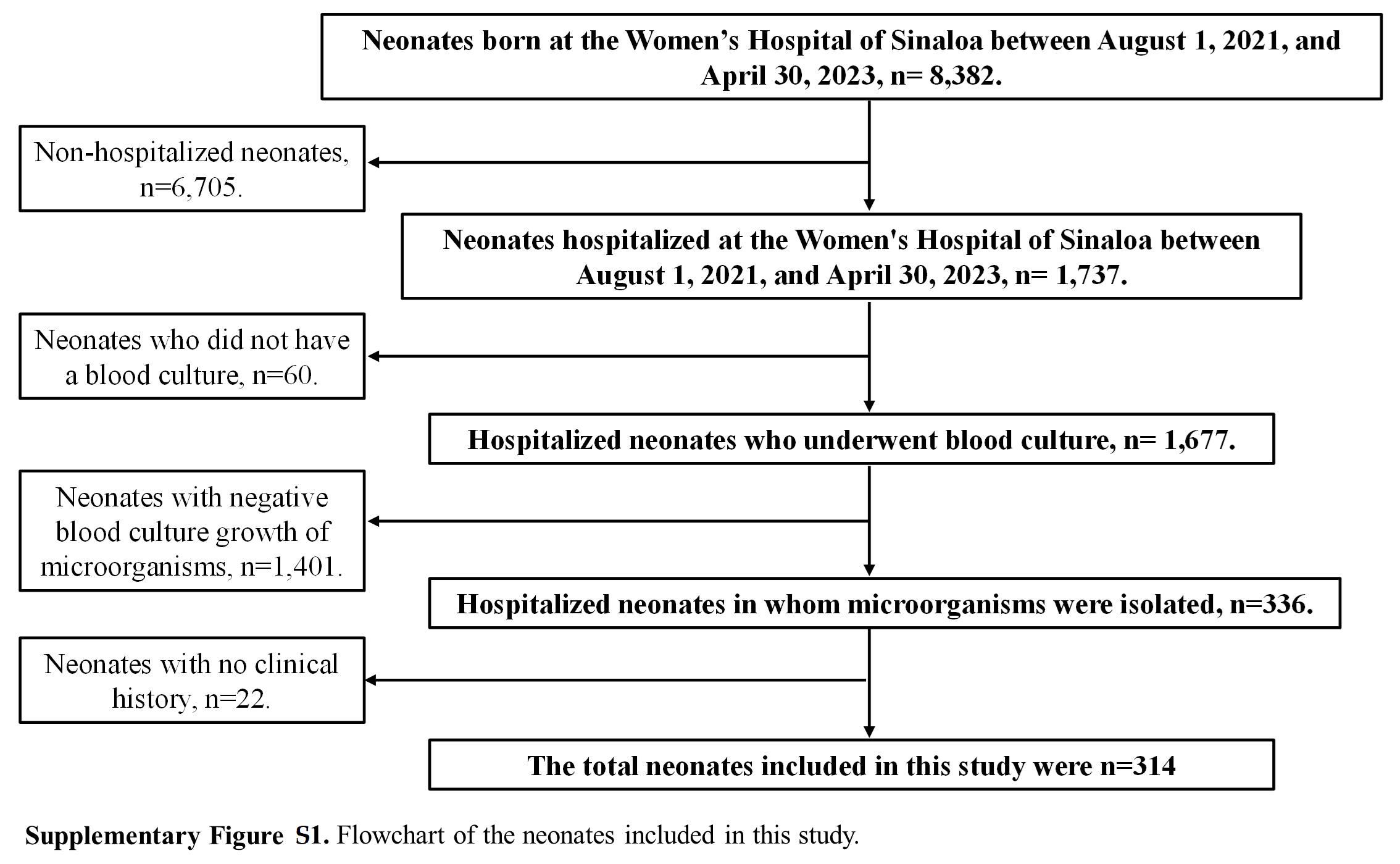

Supplement: Supplementary file 1 [file pathogens-14-00588-s001.zip › Supplementary Figure S1.tif]

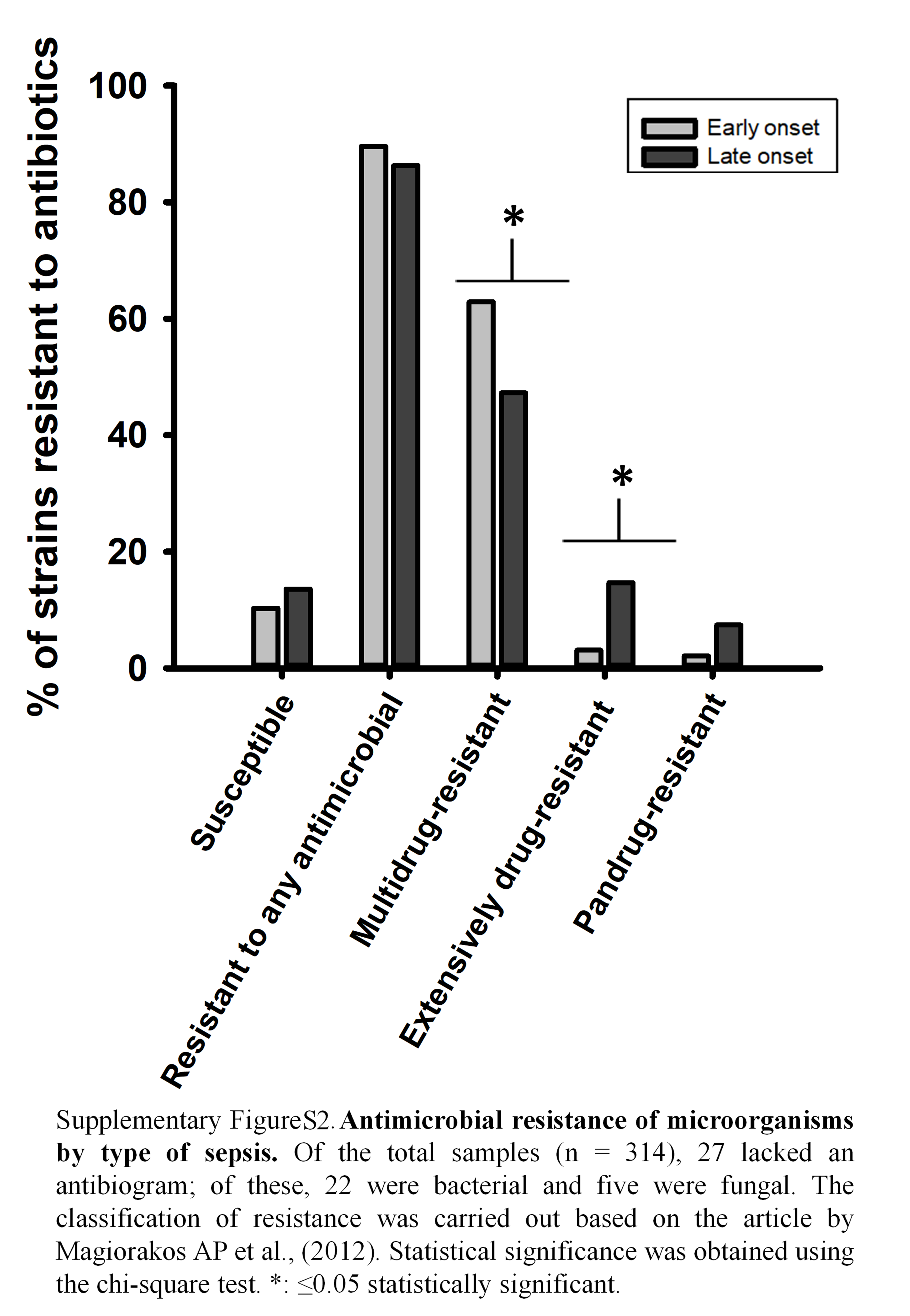

Supplement: Supplementary file 1 [file pathogens-14-00588-s001.zip › Supplementary Figure S2.tif]
